# Epicardial adipose tissue is associated with left atrial volume and fibrosis in patients with atrial fibrillation

**DOI:** 10.3389/fcvm.2022.1045730

**Published:** 2022-11-01

**Authors:** Yaacoub Chahine, Bahareh Askari-Atapour, Kirsten T. Kwan, Carter A. Anderson, Fima Macheret, Tanzina Afroze, Savannah F. Bifulco, Matthew D. Cham, Karen Ordovas, Patrick M. Boyle, Nazem Akoum

**Affiliations:** ^1^Division of Cardiology, University of Washington, Seattle, WA, United States; ^2^Department of Bioengineering, University of Washington, Seattle, WA, United States; ^3^Department of Radiology, University of Washington, Seattle, WA, United States; ^4^Institute for Stem Cell and Regenerative Medicine, University of Washington, Seattle, WA, United States; ^5^Center for Cardiovascular Biology, University of Washington, Seattle, WA, United States

**Keywords:** atrial fibrillation, epicardial adipose tissue, fibrosis, cardiac magnetic resonance imaging, Dixon sequence, late gadolinium enhancement (LGE) MRI

## Abstract

**Background:**

Obesity is a risk factor for atrial fibrillation (AF) and strongly influences the response to treatment. Atrial fibrosis shows similar associations. Epicardial adipose tissue (EAT) may be a link between these associations. We sought to assess whether EAT is associated with body mass index (BMI), left atrial (LA) fibrosis and volume.

**Methods:**

LA fibrosis and EAT were assessed using late gadolinium enhancement, and Dixon MRI sequences, respectively. We derived 3D models incorporating fibrosis and EAT, then measured the distance of fibrotic and non-fibrotic areas to the nearest EAT to assess spatial colocalization.

**Results:**

One hundred and three AF patients (64% paroxysmal, 27% female) were analyzed. LA volume index was 54.9 (41.2, 69.7) mL/m^2^, LA EAT index was 17.4 (12.7, 22.9) mL/m^2^, and LA fibrosis was 17.1 (12.4, 23.1)%. LA EAT was significantly correlated with BMI (*R* = 0.557, *p* < 0.001); as well as with LA volume and LA fibrosis after BSA adjustment (*R* = 0.579 and *R* = 0.432, respectively, *p* < 0.001 for both). Multivariable analysis showed LA EAT to be independently associated with LA volume and fibrosis. 3D registration of fat and fibrosis around the LA showed no clear spatial overlap between EAT and fibrotic LA regions.

**Conclusion:**

LA EAT is associated with obesity (BMI) as well as LA volume and fibrosis. Regions of LA EAT did not colocalize with fibrotic areas, suggesting a systemic or paracrine mechanism rather than EAT infiltration of fibrotic areas.

## Introduction

Many clinical and genetic risk factors are associated with the risk of developing atrial fibrillation (AF). In particular, the association of obesity and AF has been well established (1, 2). Obese patients have a 49% increased relative risk of developing AF compared to their non-obese counterparts (3). Animal studies have shown that obesity leads to increased atrial fibrosis and electrophysiological changes that favor the development of AF (4–6). However, the overall roles of obesity and visceral fat in AF pathophysiology in humans have not been fully elucidated. Left atrial (LA) enlargement is associated with obesity and correlates with AF risk (7). Epicardial adipose tissue (EAT), a type of visceral fat, has also been associated with AF (8, 9). It has been postulated that EAT leads to AF *via* structural and electrical remodeling of the atria and that this remodeling is modulated both directly, *via* adipocyte infiltration into the underlying atrial myocardium (4), and indirectly, *via* paracrine modulators of myocardial inflammation, oxidative stress and associated fibrosis (10). It has also been suggested that adipokines such as growth factors (e.g., tumor growth factor β1), matrix metalloproteinases and activin A released from fat contribute to fibrosis and structural remodeling of the adjacent myocardium (11–13). Thus, there is a growing interest in the delineation and quantification of EAT, its relationship with atrial fibrosis, a major factor in the pathogenesis of AF (14) and the role of EAT as a mediator of fibrosis (11, 15).

Cardiac magnetic resonance imaging (MRI) allows non-invasive volumetric quantification of epicardial fat, through fat-water separation protocols like the Dixon sequence (16). Late gadolinium enhancement (LGE) MRI can accurately assess LA myocardial fibrosis (17–19), tissue characterization of the LA wall on LGE MRI correlates well with histology from surgical biopsy specimens (20). The extent of LGE fibrosis is also associated with poor outcomes following AF ablation (21). Recently, the same method of fibrosis quantification has been implemented the DECAAF II study, a multicenter randomized clinical trial that recruited 843 persistent AF patients to compare MRI-guided fibrosis ablation plus pulmonary vein isolation vs. pulmonary vein isolation alone (22).

We aimed to assess the relationship of EAT with body mass index (BMI), LA volume and LA fibrosis in patients with AF. Then we used image processing methods to evaluate the spatial distribution of LA EAT and LA fibrosis. Our hypothesis was that EAT is an intermediary between body obesity and remodeling including atrial volume and fibrosis.

## Methods

### Study design and population

This is an observational study that enrolled 103 patients with AF undergoing cardiac MRI at the University of Washington Medical Center (Seattle, WA), prior to their first catheter ablation procedure. This study was approved by the Institutional Review Board (IRB) of the University of Washington and all participants provided verbal consent (HSD#6058). Exclusion criteria for AF patients included prior catheter ablation, implantable electric devices, severe claustrophobia, renal dysfunction and other contraindications to MRI or gadolinium-based contrast. Persistent AF status was determined using standard Heart Rhythm Society consensus criteria (23). Comorbidities and medications of the initial visit were determined using electronic medical record review. Patients with symptoms of obstructive sleep apnea underwent polysomnography to confirm the diagnosis, all patients were screened for suggestive symptoms. Study data were collected and managed using the REDCap system hosted at the University of Washington (24, 25).

### MRI protocol

All images were obtained using a Philips Medical System Ingenia 1.5 T clinical scanner. For fibrosis evaluation, LGE-MRI was acquired following the methods previously described (21). Briefly, scans were performed 15–25 min after contrast injection, using a 3D inversion-recovery, respiration-navigated, ECG-gated, gradient echo pulse sequence. Acquisition parameters included transverse imaging volume with a voxel size of 1.25 x 1.25 x 2.5 mm (reconstructed to 0.625 x 0.625 x 1.25 mm).

To assess EAT, a 3D respiration-navigated, ECG-gated Dixon sequence was obtained with the following parameters: 1.5 mm slice thickness, repetition time (TR) = 5.4 ms, echo time 1 (TE1) / echo time 2 (TE2) = 1.8/4.0 ms, flip angle (α) = 15°, voxel size = 1.5 × 1.5 × 3.0 mm^3^ (reconstructed to 1.0 × 1.0 × 1.5 mm^3^), parallel imaging factor (SENSE) = 1.5 in both phase encoding directions and water fat shift = 0.16 pixel. Arrhythmia rejection was applied, the T2 preparation duration was 50 ms and the acquisition window was 100 to 156 ms.

### Image analysis

**Figure 1:**
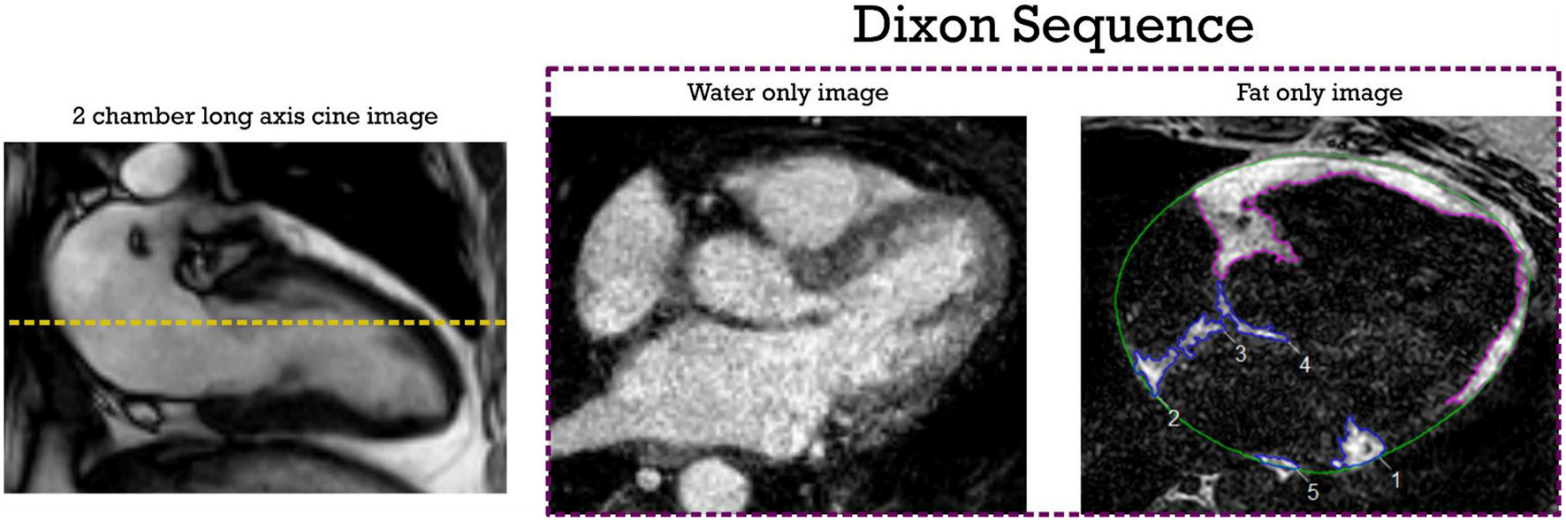
EAT segmentation from axial Dixon MRI sequence. The dotted yellow line shows the measurement level in the LA. High intensity signal areas around LA (inferior pulmonary vein level in this example) are manually contoured in blue using point by point contour tools. Ventricular EAT is contoured in purple; Total epicardial fat is calculated by adding atrial and ventricular EAT across all slices. The pericardial outline is shown in green. EAT, epicardial adipose tissue; LA, left atrial.

Pre-ablation LGE-MRI based fibrosis quantification was carried out by a third-party image processing service (Merisight, Marrek Inc., Salt Lake City, UT) using previously described methods (21). Briefly, the specific procedure for LA segmentation included: first defining the endocardium of the LA, next dilating the endocardial segmentation by 2 mm and then manually editing it to create and estimate of the boundary of the epicardial LA surface, finally subtracting the endocardial segmentation from the epicardial segmentation to create a wall segmentation. The relative extent of fibrosis was quantified within the LA wall with a threshold-based algorithm described in detail in Oakes et al. (26). Atrial fibrosis was reported as a percentage of the LA wall volume. Supplementary Figure S1 shows the image processing steps to quantify LA fibrosis from the LGE MRI sequence of one of the study patients. The investigators analyzing and quantifying fibrotic areas in the LA were blinded to the fat sequences. Dixon images were exported on CVI42 software (Circle Cardiovascular Imaging Inc., version 5.6, Calgary, AB). EAT was defined as the adipose tissue located between the visceral layer of the pericardium and outer surface of the myocardium. Areas of fat were segmented in the axial view using the software contouring tools (Figure 1). LA EAT was characterized by high signal intensity areas around the LA in a series of slices starting from the bifurcation of the pulmonary artery to the mitral annulus craniocaudally. The pericardium was identified in axial images and used as the external border for EAT. Total EAT was calculated as sum of atrial and ventricular EAT in all slices from the pulmonary artery bifurcation to the ventricular apex.

To facilitate comparison of spatial patterns of the two tissue types, we reconstructed 3D models of LA EAT quantified from Dixon MRI. Dixon sequence image stacks were loaded into Cemrg App (https://cemrg.com/) (27) where slice-by-slice segmentation of EAT, as described above, was repeated. The approximate geometry of the LA chamber (which is dark on Dixon images) was also segmented; LA contours are not as obvious in these images as in LGE-MRI scans, but they are clear enough to ascertain the locations of the pulmonary veins, mitral valve annulus, and LA appendage, which are used as fiduciary markers in the next step. Once the segmentations were complete, Cemrg App's built-in 3D reconstruction functionality was used to export separate representations of the EAT and LA volumes in the Visualization Toolkit (VTK) format. We then used the finite element mesh generator software gmsh (https://gmsh.info/) (28) to convert the EAT reconstruction from a hollow shell structure to a volumetric representation (average tetrahedral edge length ~1 mm). The LA and EAT reconstructions from Dixon scans were then loaded into the Paraview visualization software (https://www.paraview.org/) (29) alongside the original 3D renderings of the fibrotic LA derived from LGE-MRI. Translation and rotation operations were used to align the geometries of the Dixon and LGE scans using LA landmarks as fiduciary points.

We used two approaches to quantitatively examine colocalization of epicardial fat around the heart with LA regions delineated as fibrotic. First, for every point on the LA surface, we measured the minimal distance (d_EAT_) to the nearest point within the EAT volume. Second, for every point on the LA surface, we calculated the volume of EAT (V_EAT_) within a radius of 5 mm. For both metrics, we subdivided the LA surface points as either non-fibrotic or fibrotic based on LGE segmentation, and reported on differences in d_EAT_ and V_EAT_ between those regions.

### Statistical analysis

Continuous variables are reported as mean ± SD or median and interquartile ranges depending on normality testing. Normal distribution was assessed by the Shapiro-Wilk test. Categorical variables are expressed as percentages. Linear regression analysis was applied to assess three components of our hypothesis: the relationship between (1) LA and total EAT with body mass index (BMI), (2) LA EAT and LA volume, (3) LA EAT and LA fibrosis. Spearman's correlation coefficient was used to assess the relationship between continuous variables. Multivariable linear analysis was conducted to determine the independent predictors of LA volume and LA fibrosis. Variables with *p* < 0.025 in univariable analysis were entered as covariates in the multiple linear regression models. The Mann Whitney *U*-test was used to compare the minimal distance to EAT and the depth of EAT depots between fibrotic and non-fibrotic points on the LA wall. All statistical tests were 2-tailed. A *p* < 0.05 was considered to indicate statistical significance. Statistical analysis was performed using SPSS (version 22.0, International Business Machines Inc) and R Statistical Software version 4.1.1 (R Foundation for Statistical Computing) (30).

## Results

### Baseline characteristics

The median age of the cohort was 67 years (73% male). The median BMI was 28.1 (25, 34.2) kg/m^2^. The majority of our population had paroxysmal AF (64.1%). The median LA volume was 116.1 (73.8, 161.1) cm^3^ while the fibrosis burden was 17.1 (12.4, 23.1)% of the LA wall. The median LA EAT volume was 36.1 (24.5, 49.9) mL while the LA EAT adjusted to the body surface area (BSA) was 17.4 (12.7, 22.9) mL/m^2^. The mean total EAT volume was 176.2 ± 63.3 mL while the BSA-adjusted total EAT was 82.3 ± 25.2 mL/m^2^. The intraclass correlation coefficients for interobserver and intraobserver reliability of EAT measurements were 0.93 (95% CI 0.76–0.98) and 0.97 (95% CI 0.89–0.99), respectively. LA EAT was predominantly located around the posterior and inferior LA and along the atrioventricular groove. Other baseline findings are summarized in Table 1.

**Table 1:** Baseline characteristics of the 103-patient study cohort.

**Baseline characteristics**	
Age, years	67 (58, 72)
Male sex, n (%)	75 (72.8)
BMI, kg/m^2^	28.1 (25, 34.2)
BSA, m^2^	2.1 ± 0.3
Height, cm	179 (170, 185)
Weight, kg	90.7 (74.3, 109.1)
Hypertension, n (%)	53 (51.5)
CAD, n (%)	27 (26.2)
Smoking, n (%)	Former 29 (28.2) Current 7 (6.8)
CHF, n (%)	25 (24.3)
Stroke, n (%)	9 (8.7)
Obstructive Sleep Apnea, n (%)	25 (24.3)
Hyperlipidemia, n (%)	51 (49.5)
Statin use, n (%)	51 (49.5)
DM, n (%)	18 (17.5)
Paroxysmal AF, n (%)	66 (64.1)
**MRI parameters**	
LA volume, mL	116.1 (83.8, 161.1)
LA volume index, mL/m^2^	54.9 (41.2, 69.7)
LA EAT volume, mL	36.1 (24.5, 49.9)
LA EAT index, mL/m^2^	17.4 (12.7, 22.9)
Total EAT volume, mL	176.2 ± 63.3
Total EAT index, mL/m^2^	82.3 ± 25.2
LA Fibrosis, %	17.1 (12.4, 23.1)
SR during scan, n (%)	47 (45.6)

BMI, body mass index; BSA, body surface area; CAD, coronary artery disease; CHF, congestive heart failure; DM, diabetes mellitus; EAT, epicardial adipose tissue; LA, left atrial; SR, sinus rhythm.

### Association between LA and total EAT volume with BMI

**Figure 2:**
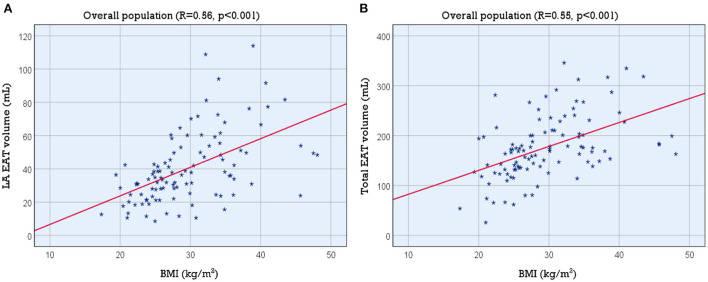
Association of LA and total EAT volume with BMI. Scatter plots showing the association of BMI with **(A)** LA EAT volume and **(B)** total EAT volume. BMI, body mass index; EAT, epicardial adipose tissue; LA, left atrial.

Linear regression demonstrated a positive correlation between LA EAT and BMI ([LA EAT] = 1.72 x [BMI]−10.55; *p* < 0.001). The strength of the association was moderate (*R* = 0.557, *R*^2^ = 0.310). Similarly, total EAT was also correlated with BMI ([Total EAT] = 4.79 x [BMI] + 34.56; *p* < 0.001, *R* = 0.545, *R*^2^ = 0.297). Figure 2 shows the corresponding scatter plots and best fit lines.

### Association between LA EAT and LA volume

**Figure 3:**
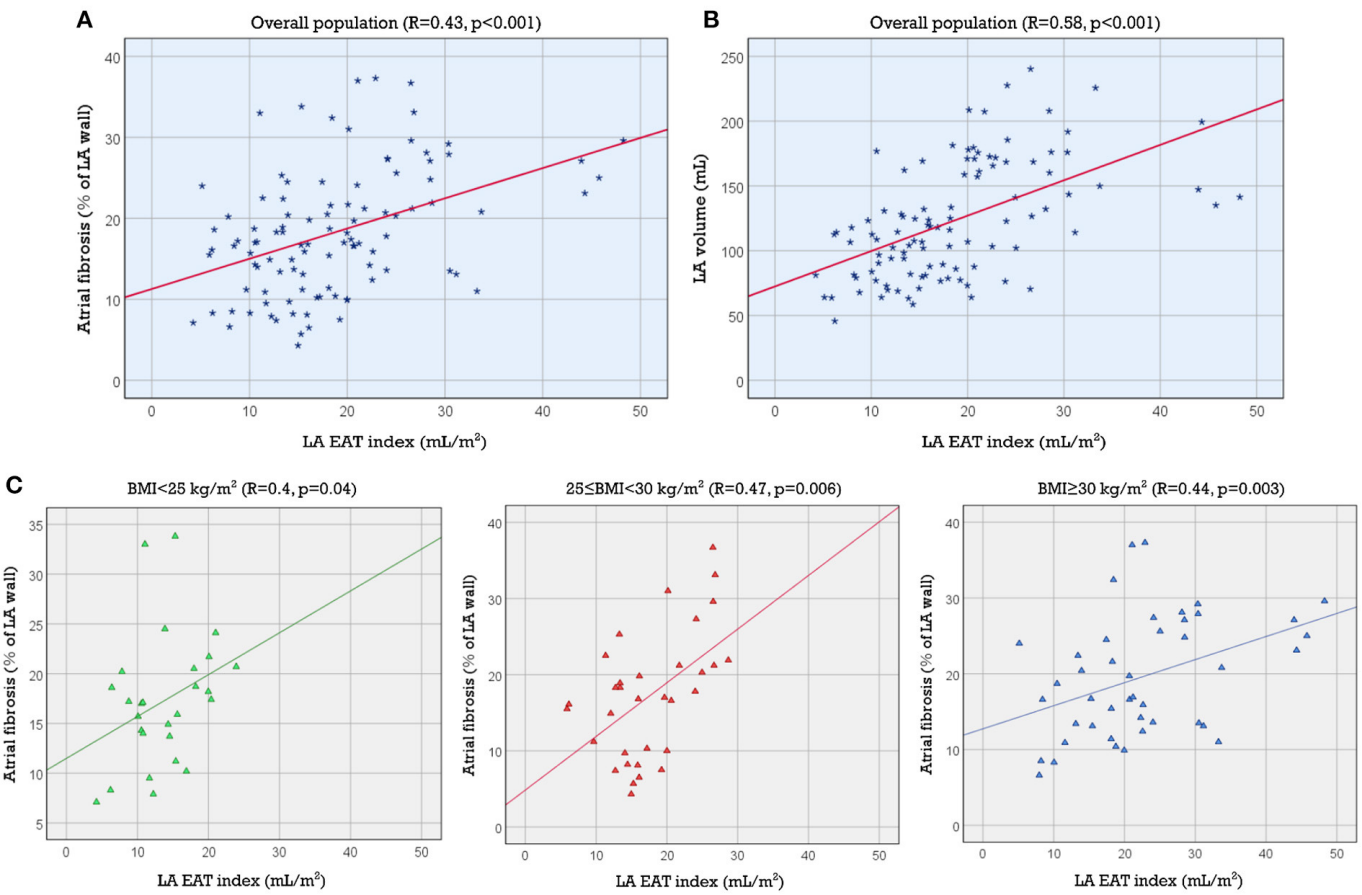
Association of LA EAT index with LA volume and fibrosis. Scatter plots showing the association between LA EAT index and LA volume in the study population **(A)** and the association between LA EAT index and LA fibrosis **(B)** in the study population and **(C)** in each of the 3 BMI categories (normal, overweight and obese). BMI, body mass index; EAT, epicardial adipose tissue; LA, left atrial.

We found a moderate correlation between LA volume and LA EAT volume (*R* = 0.578, *R*^2^ = 0.334, *p* < 0.001). The positive correlation persisted when we normalized LA EAT to BSA (*R* = 0.579, *R*^2^ = 0.335, *p* < 0.001) ([LA volume] = 2.73 x [LA EAT index] + 72.43). The scatter plot and best fit line are shown in Figure 3A.

Univariable and multivariable linear regression analyses of the association between LA volume and baseline characteristics are summarized in Table 2. Age, BMI, BSA, hypertension, congestive heart failure, persistent AF, LA EAT index, total EAT index and LA fibrosis % were each univariably associated with LA volume. LA EAT index and persistent AF were independently associated with LA volume (b coefficient, 1.58; 95% CI, 0.49–2.67, *p* = 0.005 and b coefficient 25.7; 95% CI, 9.61–41.8, *p* = 0.002, respectively). The multivariable model had an adjusted *R*^2^ = 0.448 and *p* < 0.001. LA EAT and not total EAT or systemic adiposity (BMI) was found to be independently associated with LA volume.

**Table 2:** Univariable and multivariable linear regression for the association between LA volume and baseline variables.

	**Univariable linear regression**		**Multivariable linear regression**	
	**b (95% CI)**	***p*** **-value**	**b (95% CI)**	***p*** **-value**
Age (years)	1.26 (0.44 to 2.07)	0.003	0.43 (−0.29 to 1.16)	0.239
BMI (kg/m^2^)	1.57 (0.25 to 2.89)	0.02	0.21 (−1.01 to 1.44)	0.729
BSA (m^2^)	39.68 (11.55 to 67.81)	0.006		
Sex	−19.19 (−38.5 to 0.12)	0.051		
Hypertension	17.56 (0.39 to 34.73)	0.045		
Coronary artery disease	4.06 (−15.83 to 23.95)	0.686		
Congestive heart failure	38.72 (19.79 to 57.66)	<0.001	12.2 (−5.76 to 30.17)	0.181
Obstructive sleep apnea	4.5 (−15.9 to 24.9)	0.663		
Stroke	25.01 (−0.35 to 50.37)	0.053		
Hyperlipidemia	9.98 (−7.42 to 27.38)	0.258		
Diabetes mellitus	3.06 (−13.89 to 20.02)	0.721		
Statin use	9.33 (−8.08 to 26.74)	0.290		
Smoking	8.03 (−6.1 to 22.16)	0.262		
AF type, non-paroxysmal	46.1 (30.29 to 61.92)	<0.001	25.7 (9.61 to 41.8)	0.002
LA EAT index (mL/m^2^)	2.73 (1.88 to 3.59)	<0.001	1.58 (0.49 to 2.67)	0.005
Total EAT index (mL/m^2^)	0.5 (0.17 to 0.83)	0.004	−0.02 (−0.34 to 0.3)	0.889
LA fibrosis (%)	2.46 (1.43 to 3.49)	<0.001	0.92 (−0.09 to 1.92)	0.073

AF, atrial fibrillation; BMI, body mass index; BSA, body surface area; EAT, epicardial adipose tissue; LA, left atrial.

### Association between LA EAT and LA fibrosis

Linear regression also demonstrated a statistically significant association between LA EAT volume and LA fibrosis (*R* = 0.390, *p* < 0.001). The correlation coefficient increased slightly when we normalized LA EAT to BSA (*R* = 0.432, *p* < 0.001) ([Atrial fibrosis %] = 0.37 x [LA EAT index] + 11.28). The coefficient of determination was *R*^2^ = 0.187, indicating that LA EAT could not be considered as a single predictor of LA fibrosis. The correlation between LA EAT index and LA fibrosis improved slightly in the overweight (25 ≤ BMI < 30 kg/m^2^) and obese categories (BMI ≥ 30 kg/m^2^) (*R* = 0.469, *p* = 0.006; and *R* = 0.441, *p* = 0.003, respectively) indicating that the LA EAT may have a greater effect on fibrosis in these subsets of patients. The corresponding scatter plots and best fit lines are shown in Figures 3B,C.

Univariable and multivariable linear regression analyses of the association between LA fibrosis and baseline characteristics are summarized in Table 3. Age, hyperlipidemia, persistent AF, LA EAT index, total EAT index and LA volume index were each univariably associated with LA fibrosis, whereas LA EAT index was independently associated with LA fibrosis (b coefficient 0.24; 95% CI, 0.04–0.44, *p* = 0.019). The multivariable model had an adjusted *R*^2^ = 0.256 and *p* < 0.001. Obstructive sleep apnea was not associated with LA fibrosis in our study population (*p* = 0.423).

**Table 3:** Univariable and multivariable analysis for the association of LA fibrosis with baseline variables.

	**Univariable linear regression**		**Multivariable linear regression**	
	**b (95% CI)**	***p*** **-value**	**b (95% CI)**	***p*** **-value**
Age (years)	0.18 (0.04 to 0.33)	0.013	0.05 (−0.09 to 0.2)	0.462
BMI (kg/m^2^)	0.06 (−0.17 to 0.3)	0.603		
BSA (m^2^)	2.05 (−3 to 7.1)	0.422		
Sex	−0.44 (−3.85 to 2.98)	0.8		
Hypertension	1.49 (−1.54 to 4.51)	0.332		
Coronary artery disease	1.62 (−1.82 to 5.06)	0.352		
Congestive heart failure	3.35 (−0.14 to 6.83)	0.059		
Obstructive sleep apnea	1.43 (−2.1 to 4.97)	0.423		
Stroke	1.28 (−3.2 to 5.75)	0.573		
Hyperlipidemia	2.98 (0.001 to 5.96)	0.05		
Diabetes mellitus	−1.17 (−4.1 to 1.77)	0.432		
Statin use	1.02 (−2.01 to 4.05)	0.506		
Smoking	0.73 (−1.73 to 3.19)	0.558		
AF type, non-paroxysmal	5.36 (2.38 to 8.35)	0.001	2.42 (−0.72 to 5.57)	0.13
LA EAT index (mL/m^2^)	0.37 (0.21 to 0.53)	<0.001	0.24 (0.04 to 0.44)	0.019
Total EAT index (mL/m^2^)	0.07 (0.01 to 0.13)	0.016	0.004 (−0.06 to 0.07)	0.891
LA volume index (mL/m^2^)	0.14 (0.08 to 0.21)	<0.001	0.07 (−0.01 to 0.15)	0.089

AF, atrial fibrillation; BMI, body mass index; BSA, body surface area; EAT, epicardial adipose tissue; LA, left atrial.

### Association of total EAT index with LA volume and fibrosis

Total EAT index correlated weakly with LA fibrosis (*R* = 0.24, *p* = 0.016) and LA volume (*R* = 0.28, *p* = 0.004). Supplementary Figure S2 shows the corresponding scatter plots and best fit lines. Multiple linear regression (Tables 2, 3) showed that total EAT index is not an independent predictor of either LA fibrosis or LA volume as opposed to LA EAT index (for LA volume: total EAT index b coefficient −0.02, 95% CI −0.34 to 0.3, *p* = 0.889 whereas LA EAT index b coefficient 1.58, 95% CI 0.49 to 2.67, *p* = 0.005; for LA fibrosis: total EAT index b coefficient 0.004, 95% CI −0.06 to 0.07, *p* = 0.891, whereas LA EAT index b coefficient 0.24, 95% CI 0.04 to 0.44, *p* = 0.019).

## Spatial analysis

**Figure 4:**
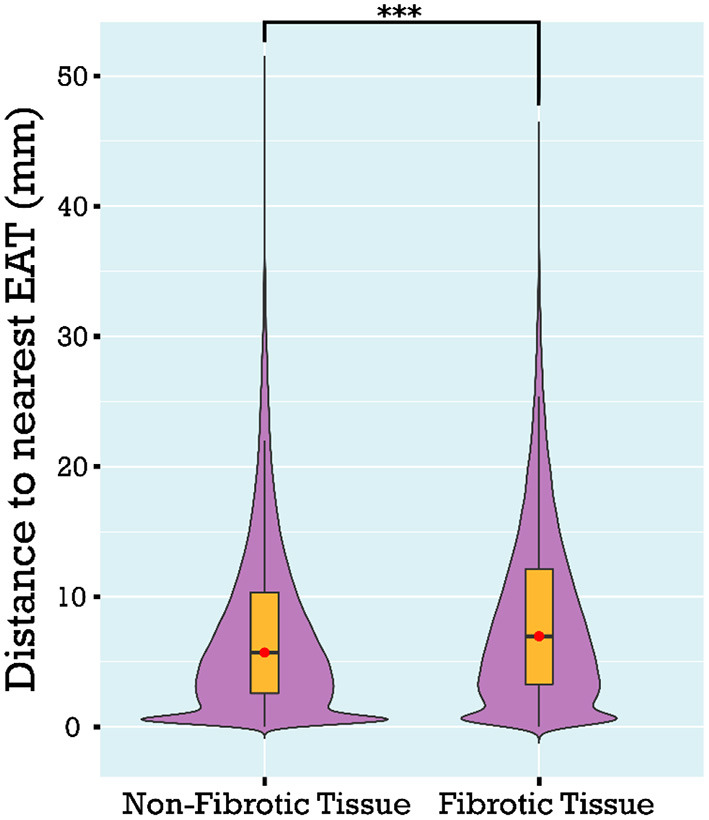
Comparison of the distance to nearest EAT for fibrotic vs. non-fibrotic areas using a violin plot showing the median and interquartile ranges. Distance to nearest EAT was shorter for non-fibrotic compared to fibrotic points on the LA surface. EAT, epicardial adipose tissue; LA, left atrial (***p < 0.001).

**Figure 5:**
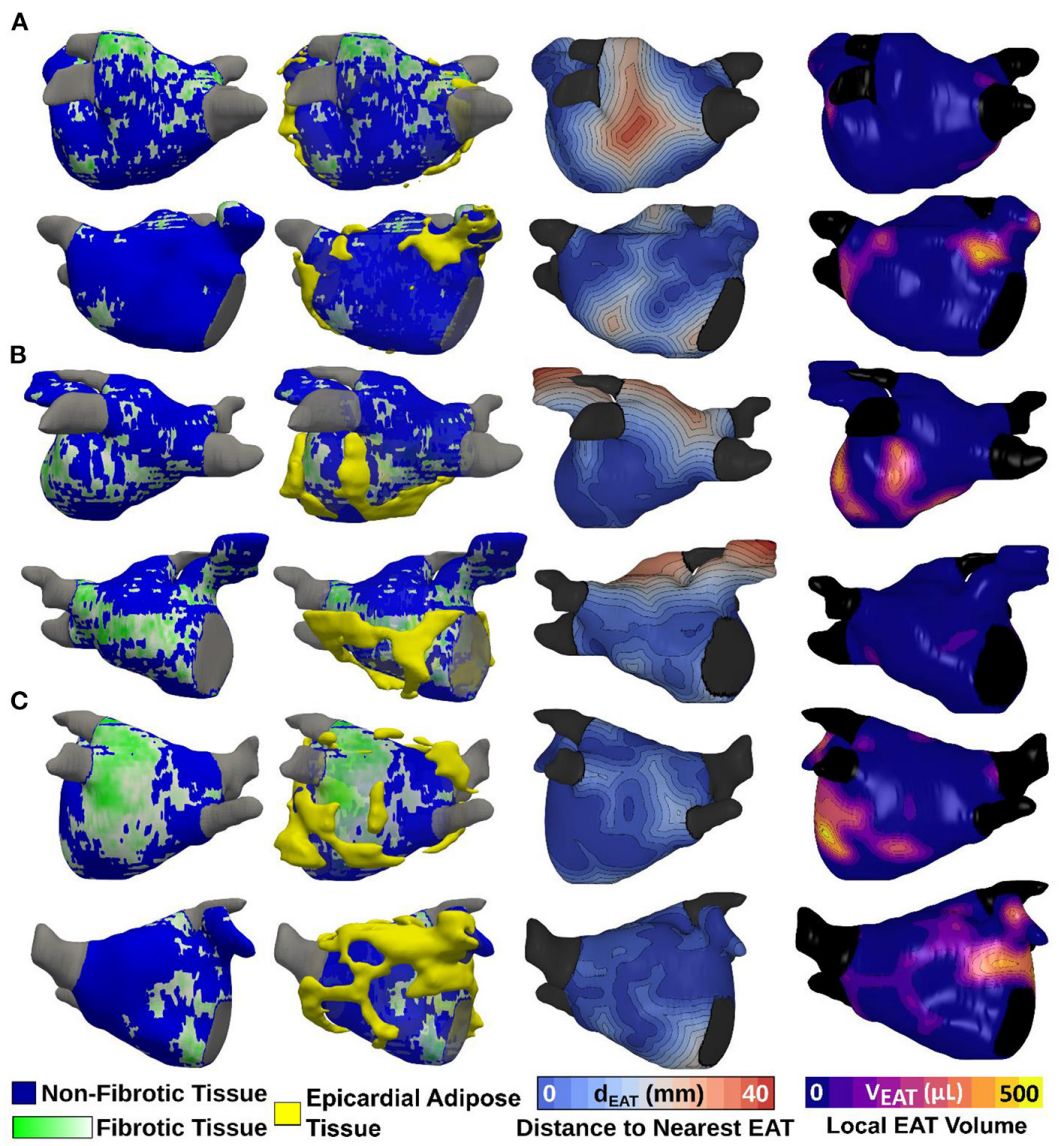
Spatial distribution of LA EAT and fibrosis, including maps of local EAT proximity and maps of local EAT volume for three different patients. 3D atrial models showing LGE fibrosis (first column) with superimposed 3D renderings of EAT (second column) are shown. Maps of local EAT proximity and local EAT volume are shown in the third and fourth column, respectively. The patient in **(A)** is an example of overlap between EAT and non-fibrotic areas. The patient in **(B)** shows more overlap between EAT and fibrotic areas whereas the patient in **(C)** demonstrates no distinct pattern of overlap. EAT, epicardial adipose tissue; LA, left atrial; LGE, late gadolinium enhancement.

As described in Methods, 3D atrial models were derived from LGE-MRI and Dixon sequence images for each patient and used to assess the extent of spatial colocalization between EAT and fibrosis. Maps of distance to the nearest region of EAT (i.e., local EAT proximity) were reconstructed and values corresponding to non-fibrotic and fibrotic parts of the LA surface were delineated. Aggregation of local EAT proximity values in all models revealed that the minimum distance to fat was significantly greater for fibrotic LA surface regions compared to non-fibrotic areas [6.966 mm (3.279, 12.127) vs. 5.714 mm (2.576, 10.347), respectively, *p* < 0.001, Figure 4]. To examine the relationship between local EAT depth (i.e., extent of EAT volume) and fibrotic remodeling, maps of V_EAT_ were reconstructed, as described in Methods. We noticed that the proportion of LA surface points having fat within a radius of 5 mm was greater for non-fibrotic areas compared to fibrotic regions [37.6% (23.4, 48) vs. 41.6% (31.2, 58.1), respectively, *p* = 0.021]. As such, we conclude that there was no predilection for spatial colocalization between EAT and fibrotic LA regions. Figure 5 shows the spatial distribution of fat and fibrosis, including maps of d_EAT_ and V_EAT_, for three representative cases. Supplementary Video S1 shows 360° views of fat, fibrosis, and LA anatomy for three individuals.

## Discussion

**Figure 6:**
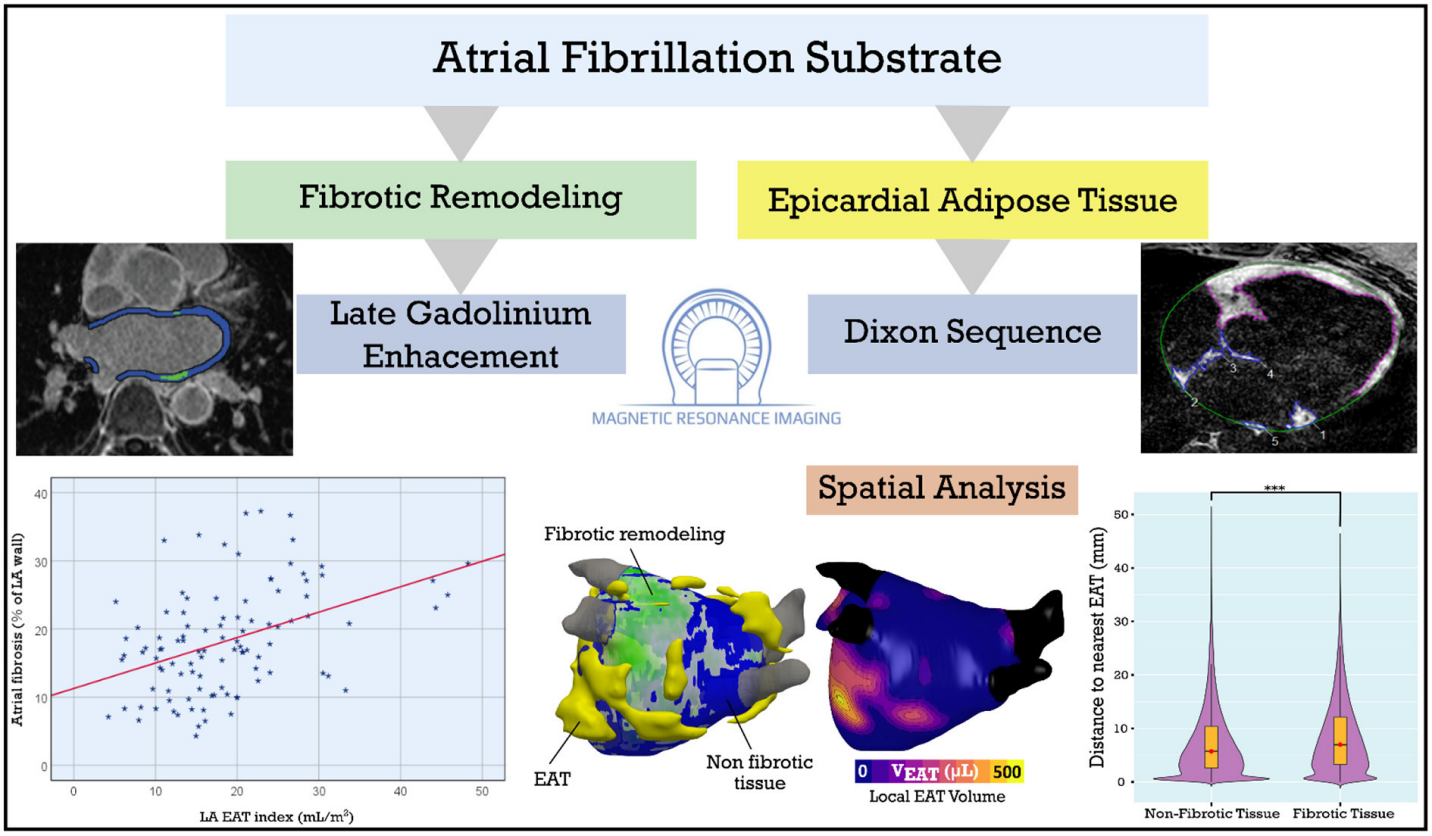
Central illustration: LA EAT and fibrosis in patients with AF. LA fibrosis and EAT, assessed using late gadolinium enhancement and Dixon MRI sequences, are positively correlated. We derived 3D models incorporating LA fibrosis and EAT to assess spatial colocalization. Spatial analysis showed no predilection for overlap between EAT and fibrotic remodeling suggesting a systemic or paracrine mechanism rather than EAT infiltration of fibrotic areas. AF, atrial fibrillation; EAT, epicardial adipose tissue; LA, left atrial. ***p < 0.001.

The major findings of the present study are as follows: (1) both LA EAT and total EAT are positively correlated with BMI, (2) LA EAT correlates well with LA volume and LA fibrosis, (3) there is no significant spatial overlap between fibrotic areas on the LA surface and pockets of EAT surrounding the heart (Figure 6, central illustration).

Obesity has been shown to be associated with epicardial fat, with a significant correlation between total EAT weight and body weight (31). Previous studies have demonstrated that BMI and pericardial fat independently increase the risk of developing AF (8, 32). EAT has also been linked to increased atherosclerotic risk (33), ventricular dysfunction (34, 35) and coronary artery disease (36). Thus, EAT may be a crucial factor mediating cardiovascular disease in obesity.

Recent studies in histological samples examined the role of EAT in myocardial fibrotic remodeling as a substrate for AF. Abe et al. (37) demonstrated that fibrotic remodeling and cytokine levels in LA EAT were associated with fibrosis burden using LA appendage samples. YKL-40, a novel biomarker for inflammation and fibrotic remodeling, is highly expressed in the EAT of AF patients; it is affected by BMI and associated with atrial fibrosis (38). EAT expression of connective tissue growth factor (cTGF) was shown to be an independent risk factor for AF and is highly associated with atrial fibrosis (39). Through right atrial appendage (RAA) sampling, Nalliah et al. showed that local EAT accumulation affects myocardial electrophysiology by direct fatty infiltration, myocardial disruption, local fibrosis and gap junction remodeling (40). RAA histological slices showed a correlation between infiltrating fat volume and fibrosis burden, but findings were limited by the small number of slices analyzed and the location (RAA) and cannot be generalized to the atrium as a whole. Mahajan et al. showed that obesity was associated with an increased EAT volume along with electroanatomical remodeling of the atria especially conduction slowing, fractionation of electrograms and areas of low voltage (41). In addition, high dominant frequency sites were shown to be located adjacent to EAT sites (42). In our study, we assessed the relationship between EAT and fibrosis, non-invasively, using different MR sequences obtained in the same setting.

Various approaches have been suggested for EAT quantification. The majority of clinical studies to date have used computed tomography (CT) scans to examine the association between epicardial fat depots and cardiovascular disease (43). CT allows for adipose tissue quantification using attenuation value ranges (generally −190 to −30 Hounsfield Units) (44); moreover, EAT quantification by CT typically does not require specific acquisitions, and can be done using unenhanced scans (often obtained for calcium scoring) or contrast-enhanced CT scans used for angiography (45).

Recently, there has been a growing interest in MRI-based fat quantification since it involves less radiation exposure than CT. In a study by Wong et al. (46) sequential steady-state free-precession cine sequences were acquired to assess pericardial fat, but this method did not allow for a reliable separation of fat and water. The 3D Dixon method, implemented by Homsi et al. (16), showed an excellent correlation between measured cardiac fat volumes and true fat volumes. Nakamori et al. measured epicardial fat volume using the 3D Dixon sequence and concluded that LA EAT volume is significantly higher in patients with AF vs. those without (47). We acquired Dixon as well as LGE-MRI sequences in one clinical scan which allowed for the analysis and correlations between EAT and atrial fibrosis (17). Recently, combined LGE and Dixon fat-water separation sequences have been proposed to simultaneously visualize fibrosis and EAT. Skoda et al. (48) showed a high level of intraobserver and interobserver repeatability as well as agreement with reference methods for assessment of fibrosis and EAT using the combined 3D LGE-Dixon technique. This sequence allows to shorten the time required to image fibrotic remodeling and EAT surrounding the heart.

Our analysis showed a statistically significant association between fat and fibrosis. The modest correlation (R^2^); however, suggests that EAT is not a single predictor factor of fibrosis. Multiple clinical factors have also been associated with fibrosis. Congestive heart failure can lead to fibrosis through increased LA stretch and neurohormonal activation through the renin-angiotensin-aldosterone axis (49). Likewise, LA pressure or volume overload caused by a valvular heart disease can lead to structural changes in the LA leading to myocardial fibrosis (50). Demographic characteristics like older age and female sex were associated with a higher fibrosis burden in AF patients (51). More than 160 genes were associated with AF during the last decades; recently, a growing body of evidence has linked structural genes with fibrosis as a substrate for AF. For example a loss of function in the MYL4 gene, which encodes an atria-specific myosin light chain, and a TTN gene mutation which codes a truncated variant of the giant sarcomeric protein titin predispose to early atrial fibrosis leading to cardiac arrhythmia (52, 53). It is currently unknown whether EAT is a marker or an active participant in these pathological relationships. Identifying individual predictors of fibrosis in each AF patient could provide better means for risk stratification and treatment of AF.

Prior work established an association between pericardial fat and LA size (32, 46) without taking into account the different subtypes of adipose tissue surrounding the heart. More recently, studies focusing on EAT imaging showed a correlation with LA volume (47, 54). Our results support this association even after adjusting the LA EAT to BSA. Since there is no separation between atrial and ventricular EAT beneath the pericardium, theoretically total EAT should affect LA volume and fibrosis, however and as shown in previous studies like Nalliah et al. (40) and Maimaituxun et al. (55) local EAT seems to exert an effect on the anatomically contiguous myocardial tissue beyond the effect of total EAT. Our results show that it is LA EAT, not total EAT or systemic obesity that is associated with LA volume and fibrosis. These results suggest that LA EAT may have an effect on LA structural remodeling in AF patients, or vice versa, but the mechanism involved is still not clear.

Our patient derived 3D atrial models showed no significant spatial overlap between EAT and fibrotic areas of the LA wall. This lack of colocalization lends support to the notion that EAT's role in the pathogenesis of myocardial fibrosis is mainly through paracrine and endocrine modulators (11), especially TGFβ1, and therefore direct spatial overlap may not be required. In addition, recent evidence suggests that direct adipocyte infiltration of the contiguous atrial myocardium leads to a slowed and inhomogeneous conduction that can act as a substrate of AF independently of fibrosis, as shown in both animal (4, 56) and human tissue (40). Post myocardial infarction infiltrating fat has recently been recognized as a proarrhythmic substrate that predisposes patients with ischemic cardiomyopathy to ventricular tachycardias (VT) (57, 58), and a novel computational approach has been used to predict VT ablation targets based on the pattern of infiltrating fat (59). Further research is required to determine whether AF ablation targets can also be predicted based on atrial adipose tissue infiltrates.

Our study focused on LA EAT in particular and not total atrial EAT as we aimed to assess its relationship to LA fibrosis, a well-known AF substrate, in terms of quantitative assessment and spatial analysis; whether the correlation between the two persists when studying the right atrium remains to be investigated.

Our data suggest that EAT may have a pathogenic effect on the atria beyond systemic effects of generalized adiposity. Weight loss in obese ovine models is associated with reduced EAT volume along with reversal of interstitial fibrosis and lower AF inducibility (60). Whether or not the fibrotic and anatomic remodeling caused by LA EAT in humans can be reversed or at least attenuated by reducing the patient's BMI remains to be seen. We previously confirmed the role of atrial fibrosis on AF and the use of LGE-MRI as a predictor of ablation outcome (20, 21). A potential role for EAT in predicting ablation outcomes or modulating the impact of ablation on atrial tissue requires further investigation.

## Limitations

Our study has some limitations. First, it is a single-center observational study with a relatively small study population and no control group without AF. Second, the process of fat quantification is performed manually, this could be a time-consuming process and less feasible for use in clinical practice but may be significantly facilitated through artificial intelligence and automation. Third, the image quality can vary among patients and artifacts might be mischaracterized as fat. Fourth, we recognize that multiple methods for fibrosis quantification have been described in the literature with varying results (61) and the lack of a well-established standard for quantifying fibrosis can be considered as a limitation.

## Conclusion

We have shown that elevated BMI is associated with increased EAT. Further, LA EAT is associated with LA volume independently of total EAT and systemic obesity. The analysis of data derived from MRI (LGE and Dixon sequences) and 3D models reconstructed from the same scans confirms the moderate association of LA epicardial fat and fibrosis, without evidence of spatial colocalization of the two tissue types. These findings further our understanding of the determinants of the AF substrate and support a role for LA EAT as a systemic contributor to atrial remodeling and a link between obesity and AF.

## Data availability statement

The raw data supporting the conclusions of this article will be made available by the authors, without undue reservation.

## Author contributions

YC: conceptualization, data curation, formal analysis, validation, investigation, methodology, and writing original draft. BA-A: data curation, formal analysis, validation, and writing review and editing. KK, CA, and SB: data curation, software, formal analysis, visualization, validation, and writing review and editing. FM and TA: data curation, formal analysis, visualization, validation, and writing review and editing. MC and KO: conceptualization, methodology, investigation, and writing review and editing. PB: conceptualization, software, methodology, supervision, and writing original draft, project administration, and writing review and editing. NA: conceptualization, resources, funding acquisition, supervision, investigation, methodology, writing original draft, project administration, and writing review and editing. All authors read and approved the final manuscript.

## Funding

This work was supported by the John Locke Charitable Trust to NA.

## Conflict of interest

The authors declare that the research was conducted in the absence of any commercial or financial relationships that could be construed as a potential conflict of interest.

## Publisher's note

All claims expressed in this article are solely those of the authors and do not necessarily represent those of their affiliated organizations, or those of the publisher, the editors and the reviewers. Any product that may be evaluated in this article, or claim that may be made by its manufacturer, is not guaranteed or endorsed by the publisher.

## Abbreviations

AF: atrial fibrillation
BMI: body mass index
BSA: body surface area
CT: computed tomography
EAT: epicardial adipose tissue
ECG: electrocardiogram
LA: left atrial
LGE: late gadolinium enhancement
MRI: magnetic resonance imaging.
