# Supplementary material for: Epicardial adipose tissue is associated with left atrial volume and fibrosis in patients with atrial fibrillation
Source: Front Cardiovasc Med. 2022 Nov 1;9:1045730. doi: 10.3389/fcvm.2022.1045730 (PMC9664066; doi:10.3389/fcvm.2022.1045730)
Supplement: Supplementary file 2 [file Data_Sheet_1.PDF]

Supplemental material

Epicardial Adipose Tissue Is Associated With Left Atrial Volume And Fibrosis In Patients With Atrial Fibrillation

Running title: Epicardial Adipose Tissue And Fibrosis In AF

Yaacoub Chahine, MD,[a] Bahareh Askari-Atapour, MD,[a] Kirsten T Kwan, BS,[b] Carter A Anderson, BS,[b] Fima Macheret, MD, MS,[a] Tanzina Afroze, MD,[a] Savannah F Bifulco, BS,[b] Matthew D Cham, MD,[c] Karen Ordovas, MD, MAS,[c] Patrick M Boyle, PhD,[b,d,e] Nazem Akoum, MD, MS[a,b]

[a]Division of Cardiology, University of Washington, Seattle, WA

[b]Department of Bioengineering, University of Washington, Seattle, WA

[c]Department of Radiology, University of Washington, Seattle, WA

[d]Institute for Stem Cell and Regenerative Medicine, University of Washington, Seattle, WA

[e]Center for Cardiovascular Biology, University of Washington, Seattle, WA

Supplemental methods

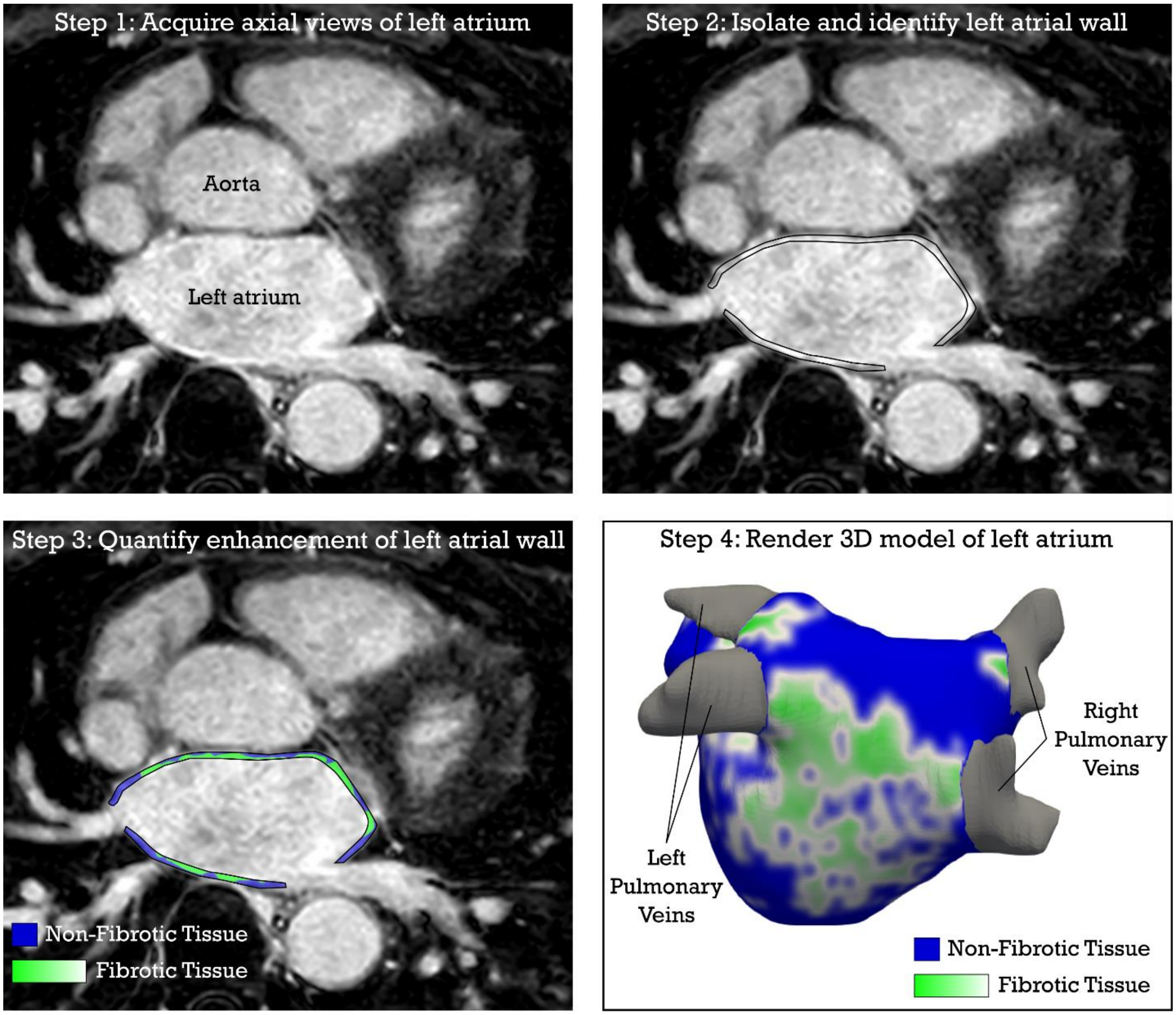

Supplemental figure 1: Atrial LGE MRI and image processing steps to quantify fibrosis. Step 1 shows atrial LGE MRI acquisition, step 2 shows left atrial wall segmentation, step 3 shows pixel mapping of fibrotic/non-fibrotic areas, step 4 shows 3D model rendering of atrial fibrosis by repeating steps on a slice-by-slice basis.

Supplemental results

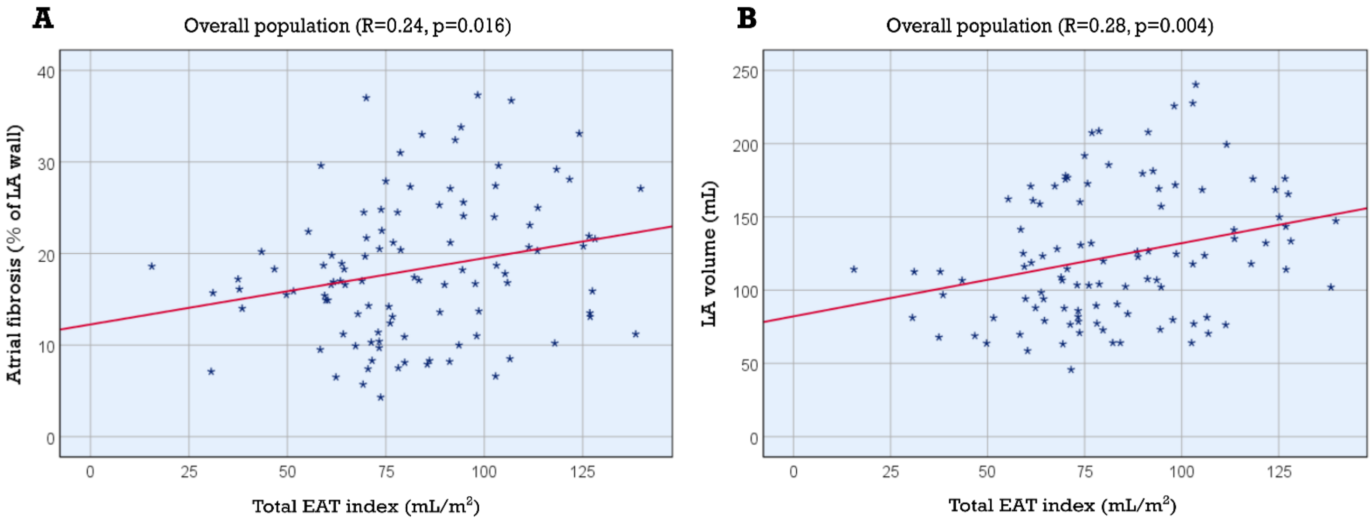

Supplemental figure 2: Association of total EAT index with LA volume and fibrosis. Scatter plots showing the association between total EAT index and LA volume (A) and the association between total EAT index and LA fibrosis (B) in the study population. EAT = epicardial adipose tissue, LA = left atrial
